# Sport Supplement Use in 14–18-Year-Old Adolescents: A Single-Group Pre–Post Social Media Educational Intervention Study

**DOI:** 10.3390/nu18121849

**Published:** 2026-06-08

**Authors:** Nikola Jojić, Mire Zloh, Nataša Jovanović Lješković, Suzana Miljković, Svetlana Stojkov, Marina Kalić, Slađana Vojvodić, Milan Ilić, Aleksandra Jovanović Galović

**Affiliations:** 1 Faculty of Pharmacy Novi Sad, University Business Academy in Novi Sad, Heroja Pinkija 4, 21000 Novi Sad, Serbia; nikola.jojic@ffns.ac.rs (N.J.); mire.zloh@ffns.ac.rs (M.Z.); natasa.ljeskovic@ffns.ac.rs (N.J.L.); suzana.miljkovic@ffns.ac.rs (S.M.); svetlana.stojkov@ffns.ac.rs (S.S.); marina.kalic@ffns.ac.rs (M.K.); sladjana.vojvodic@ffns.ac.rs (S.V.); milan.ilic@ffns.ac.rs (M.I.); 2 College of Vocational Studies for the Education of Preschool Teachers and Sports Trainers in Subotica, Banijska 67, 24000 Subotica, Serbia

**Keywords:** sport supplements, adolescents, young athletes, social media, education

## Abstract

Background: The use of sports supplements among adolescents is rising globally, driven by fitness trends and social media influence, yet knowledge gaps persist. This study aimed to assess supplement usage patterns, knowledge, attitudes, information sources, and the impact of a social media educational intervention among Serbian secondary school students. Methods: A single-group pre–post educational intervention study was conducted in secondary school students (aged 14–18) in Vojvodina, Serbia. A 21-question anonymous questionnaire was distributed to 1000 students along with parental informed consent forms. Pre-intervention survey assessed sociodemographics, physical activity and social media habits, supplement use information sources, and awareness of risks and banned substances. Based on the initial findings, an educational campaign delivered 56 short videos (≈70 s each) on Instagram and TikTok covering most frequently used supplements (e.g., creatine, proteins, caffeine, energy drinks). After, the intervention survey was repeated. The data were analyzed using the McNemar–Bowker test of symmetry. Results: In this study, 65% of Serbian secondary school adolescents reported being physically active, engaging predominantly in gym workouts and team sports. The majority of participants initiate dietary supplement use independently, without consulting healthcare professionals or adults. The most commonly used supplements were vitamins and minerals, while energy drinks ranked notably high. Social media intervention had a limited impact due to its short duration; however, certain changes were detected. Conclusions: Serbian adolescents frequently use sports supplements without adequate professional guidance. Long-term TikTok/Instagram interventions could be used in the future in order to influence behaviors and improve knowledge about sport supplement use.

## 1. Introduction

According to Directive 2002/46/EC, dietary/food supplements are foodstuffs, the purpose of which is to supplement the normal diet. They are concentrated sources of nutrients or other substances with a nutritional or physiological effect, marketed in dose form [1]. The regulatory framework for dietary supplements in Serbia is harmonized with the one applied in the European Union (EU) [2], and products for active and recreational athletes are regulated as dietary supplements.

Over the past few decades, the global consumption of dietary supplements has significantly increased [3]. The sports nutrition segment is projected to experience the fastest growth, with a compound annual growth rate (CAGR) of 7.69% between 2025 and 2030. This trend is largely driven by the rising focus on fitness and overall well-being. Furthermore, the expanding influence of athletes and fitness figures on social media platforms has further fuelled the segment’s expansion and contributed to greater visibility and use of these products [4]. Another estimate places the global sports nutrition market at USD 45.25 billion in 2023, with a projection to reach USD 75.01 billion by 2030, with a CAGR of 7.5%. Within this, sports supplements accounted for USD 17.83 billion in 2023 [5]. In Serbia, no precise national data exist for sports supplements usage rates, but Euromonitor’s national forecasts (Euromonitor International’s Sports Nutrition in Serbia report) show an important retail volume and current value growth driven by rising health and fitness awareness, especially accelerated during the COVID-19 pandemic [6]. While dietary supplement consumption—particularly among young athletes and adolescents—appears to be at an all-time high [7,8,9,10,11,12,13,14], consumer knowledge levels remain relatively low and inadequate and do not correspond to the pace of market growth [8,15].

Existing knowledge gap is especially concerning in the case of young athletes, who often lack the ability to critically evaluate the quality and credibility of information obtained from the internet, coaches, salespeople, or social media platforms [14,16,17]. The World Anti-Doping Agency (WADA), as an international authority in the field of sport substance use, has developed an array of education programs present on the Anti-Doping Education and Learning platform, ADEL “https://adel.wada-ama.org/learn (accessed on 13 January 2026)”. In the context of young athletes, special programs are developed for parents (Parents of High Performance Athletes’ Education), available in eight different languages.

Recent evidence indicates that adolescents and young adults frequently rely on social media as a source of health- and nutrition-related information. It is shown that increased social media use is associated with behavioral and cognitive patterns that may influence health-related decision-making, including susceptibility to misinformation and fear of missing out [18] as well as burnout, reduced well-being, and increased vulnerability to external influences [19,20,21]. The potential for problematic or addictive social media use further underscores the need to consider its role in shaping adolescents’ health behaviors and perceptions [22].

Taking all these data together, a need for new approach for adolescent education regarding sport supplement use is evident [23,24]. This study explores the potential effects of short-term educational intervention on social media platforms.

Our main aims were to: (i) obtain an insight into adolescents’ knowledge and practices regarding sports supplementation; (ii) identify main sources of information on this topic; and (iii) create an educational intervention on social media platforms to encourage the correct use of supplements.

## 2. Materials and Methods

Before the initiation of the project activities, institutional support was obtained from the principal educational authority of Vojvodina (Provincial Secretariat for Education, Regulations, and Administration, National Communities and National Minorities of the Autonomous Province of Vojvodina; “https://www.puma.vojvodina.gov.rs/index.php?lang=7 (accessed on 22 December 2025)”. Subsequently, an invitation letter for participation with all pertaining information was sent to all secondary schools in Vojvodina. Only the schools that officially accepted the invitation were enrolled in a project.

### 2.1. Study Design

The project was conducted as a single-group pre–post educational intervention study and included four phases:

*Phase I: Distribution of the informed consent form*

Informed consent forms were distributed to secondary school students in grades I–III. The distribution and collection of the signed informed consent forms were conducted in person. All activities were performed in collaboration with members of the school staff, including school principals, pedagogues, psychologists and physical education teachers.

*Phase II: Entry questionnaire (pre-intervention)*

A total of 1000 entry questionnaires (pre-intervention) were distributed to all participants whose parents had signed the consent form. Students anonymously filled out a pre-intervention questionnaire, which was distributed in an open coded envelope. Upon completion, participants were instructed to seal the envelope and deposit it in a collection box.

*Phase III: Social media intervention*

Based on the data collected from the entry questionnaires, platforms were created on Instagram and TikTok. The educational video material comprised 56 videos, with a duration of approximately 70 s each. Videos covered 6 topics, identified as insufficiently understood based on the entry survey data. Each topic (creatine, probiotics, caffeine, aminoacids and proteins, energy drinks, and pre/post-workout supplements) was addressed in 9–10 videos, posted over a six-month period. Educational content was developed by university professors, experts in nutrition. The content was age-appropriate, easily understandable and engaging for adolescents, without excessive technical terminology while preserving scientific accuracy.

*Phase IV: Follow-up questionnaire (post-intervention)*

The effects of this intervention were evaluated on the same group of participants who filled the entry questionnaire, by following the same procedure. After the six-month period, the follow-up questionnaire was distributed. Effects of the social media intervention were assessed by comparing participants’ answers given in the entry questionnaire (pre-intervention) and follow-up questionnaire (post-intervention). To enable individual-level pre–post pairing while preserving participant anonymity, each envelope was assigned a unique code. Statistical significance of the differences was determined using a data for the pre- and post-intervention group.

### 2.2. Questionnaires

The entry (pre-intervention) questionnaire consisted of 21 questions, divided into VI groups: (i) socio-demographic characteristics (age, gender, parental education); (ii) social media preferences; (iii) sport activities participation; (iv) knowledge and behavior related to dietary supplement use; (v) social media influence as a source of information on supplements; and (vi) awareness of side effects of dietary supplements and prohibited substances in sports. The follow-up (post-intervention) questionnaire consisted of exactly the same questions, excluding Sections 1–3 which covered general information and could have not been influenced by our educational intervention (age, gender, social network habits and sport activity). The estimated time for filling out the questionnaire was ≤15 min.

### 2.3. Limitations of the Study

The main methodological limitation of the study was the lack of a control group. Since the designed educational content was publicly accessible via social media platforms, access could not have been restricted. This fact made it impossible to have a control group. Additionally, a high attrition rate among the respondents was evident. However, it may be explained by the identical wording of pre- and post-intervention questionnaires causing response fatigue in adolescents. The anonymity of the responses, maintained through the envelope coding system, may have contributed to the attrition, as participants were not personally reminded or followed up. Although the possibility of attrition bias exists, only successfully matched pairs were included in the statistical analysis.

### 2.4. Statistical Analysis

Differences in the distribution of paired categorical data before and after education were evaluated using the McNemar–Bowker test of symmetry. The *p* value was calculated with McNemar’s test with the continuity correction. A *p*-value of less than 0.05 was considered statistically significant. Software Jamovi 2.6.44 “https://www.jamovi.org (accessed on 21 April 2026)” was used.

### 2.5. Ethical Approval

The research was approved by the Ethics Committee of the Faculty of Dentistry in Pančevo on 22 June 2021 (file number 1072/21) and the Ethics Committee of the World Anti-Doping Agency (WADA) on 5 July 2021.

## 3. Results

### 3.1. General Characteristics and Sport Supplement Use Patterns

The pre-intervention survey was completed by 808 students and the post-intervention survey by 650 students. The attrition rate (19.55%) was noted, and could be attributed to the age group of the participants. Moreover, it could be because the pre- and post-intervention questionnaires contained the same questions.

**Figure 1:**
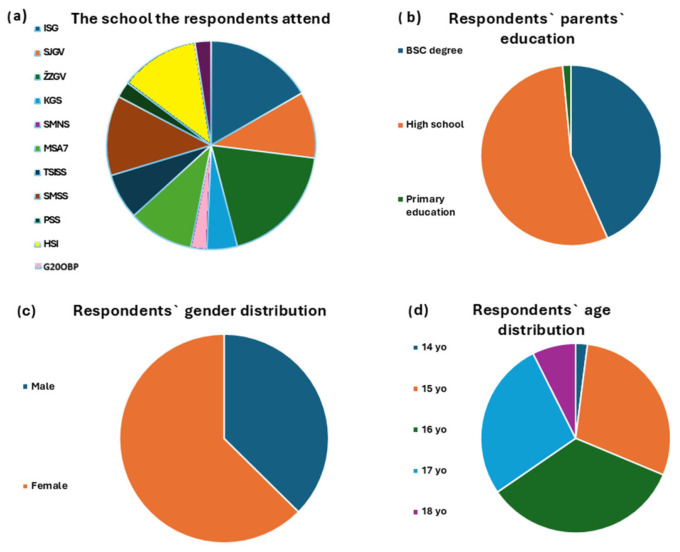
Categorisation of participants according to (**a**) age, (**b**) gender, (**c**) parents’ education, and (**d**) school they attend: ISG—Isidora Sekulić Gymnasium, SJGV—Stevan Jakovljević Gymnasium Vlasotince, ŽZGV—Žarko Zrenjanin Gymnasium Vrbas, KGS—Karlovci Grammar School, SMNS—Smart Gymnasium in Novi Sad, MSA7—Medical School April 7, TSISS—Technical School Ivan Sarić Subotica, SMSS—Secondary Medical School Subotica, PSS—Polytechnic School Subotica, HIS—High School Ivanjica, G20OBP—Gymnasium “20. oktobar” Bačka Palanka.

The first section included questions to allow for the categorisation of participants according to age, gender, level of engagement in physical activities and type of activity if relevant, as well as parental education. Participant demographics are shown in Figure 1.

Approximately 90% of participants were 15–17 years of age, with a higher proportion of female students, which may be attributed to the types of schools included in the study. Parental education was approximately equally distributed between completed secondary education and a Bachelor’s degree. Participants enrolled in this study attended a broad range of school types, including vocational, technical, and general high schools.

The selection of participating schools was not biased towards any particular educational track. This approach enabled the inclusion of a diverse and representative high school population.

**Figure 2:**
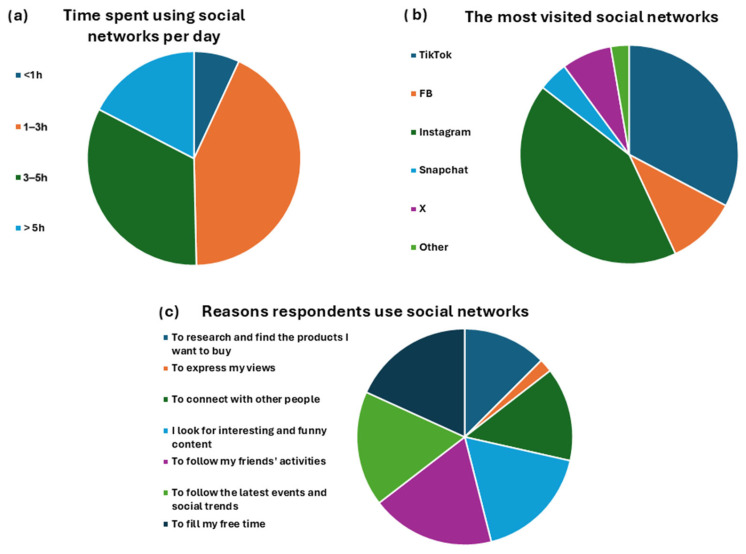
Categorisation of participants according to: (**a**) time spent using social networks per day, (**b**) social network usage, and (**c**) reasons for social networks use.

The second section of the questionnaire aimed to get insights on social media habits (Figure 2).

Approximately three-quarters of respondents reported spending more than one hour per day on social media, while over one-third indicated daily usage exceeding three hours.

Instagram and TikTok emerged as the two most frequently used platforms among participants. The reasons for using social media were diverse and relatively evenly distributed, including connecting with other people, looking for interesting and funny content, and following friends’ activities, social trends and events.

The varied and nearly evenly distributed motivations for social media use suggest that adolescents engage with these platforms for a wide range of purposes, rather than being driven by a single dominant factor. Although Instagram and TikTok were the two most frequently used platforms among respondents, TikTok proved more popular for study-related content, receiving more views and likes.

**Figure 3:**
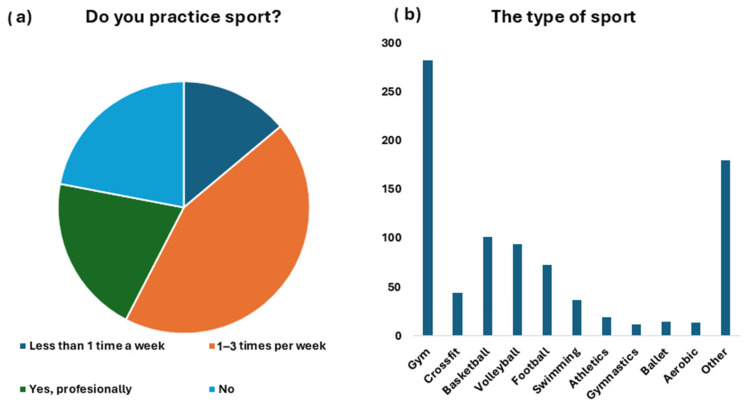
Categorisation of participants according to: (**a**) self-reported sport activity frequency, and (**b**) the types of sport.

The third section of the questionnaire was dedicated to estimating the regularity and type of physical activity (Figure 3).

Overall, approximately 60% of participants reported being physically active. Among the types of physical activity, gym workouts were the most commonly reported. The “other” category likely includes activities such as pilates and aerobics. The predominance of gym-based activities aligns with current fitness trends among youth, while the presence of pilates and aerobics in the “other” category likely reflects a growing interest in structured, non-competitive forms of exercise. Although traditional team sports such as football, basketball, and volleyball were reported less frequently, their continued presence confirms their ongoing cultural relevance in Serbia.

Sections 4 to 6 of the questionnaires comprised the questions that were present both in entry survey (pre-intervention), and follow-up survey (post-intervention). The results are shown together, for comparison.

**Figure 4:**
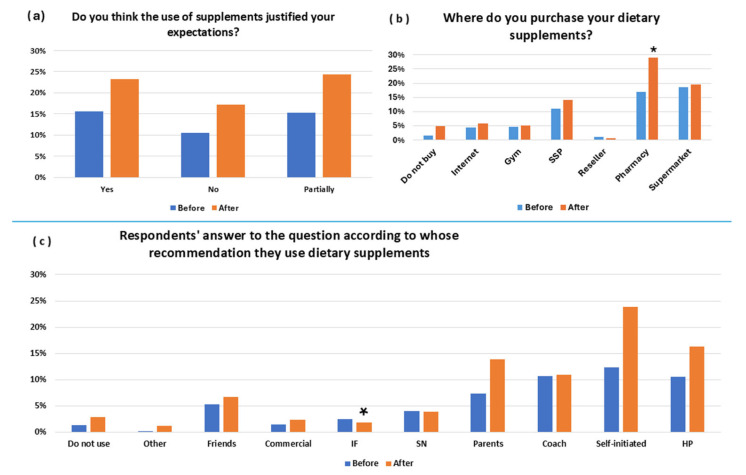
(**a**) Distribution of participant opinions regarding whether the use of supplements justified their expectations. (**b**) Distribution of participants by reported sources for purchasing dietary supplements (SSP—specialized supplement shops). (**c**) Sources of influence reported by participants when deciding to use dietary supplements (IF—internet forums, SN—social networks, HP—healthcare professionals, doctor/pharmacist/nutritionist). Completed questionnaires before (808), and completed questionnaires after the intervention (650). Values marked by asterisks (*) indicate statistically significant differences (*p* ˂ 0.01).

The fourth part of our questionnaire consisted of questions dedicated to supplement use, guidance and the expected effects (Figure 4).

Approximately half of supplement users reported that dietary supplements met their expectations to a moderate or high degree, combining “Yes” (~16% before, ~23% after) and “Partially” (~15% before, ~24% after) responses. “No” responses were observed in ~10% before and ~17% after the intervention. Overall, this perception did not change significantly following the intervention.

Pharmacy was the most commonly reported purchase source both before (~17%) and after (~28%) the intervention, with this increase being statistically significant (*p* < 0.01, odds ratio = 1.591; CI = 1.076–2.375). Supermarket purchases remained stable (before ~18%, after ~20%), while SSP increased from ~11% to ~14%. Modest increases were also noted for “Do not buy” (~3% to ~5%) and “Internet” (~4% to ~6%), whereas “Gym” showed a slight decline (~5% to ~4%). The statistically significant shift toward pharmacy as a purchase source has been recorded.

The greater self-initiation of information-seeking on dietary supplements and increased parental involvement is visible (Figure 4c), but not statistically significant.

**Figure 5:**
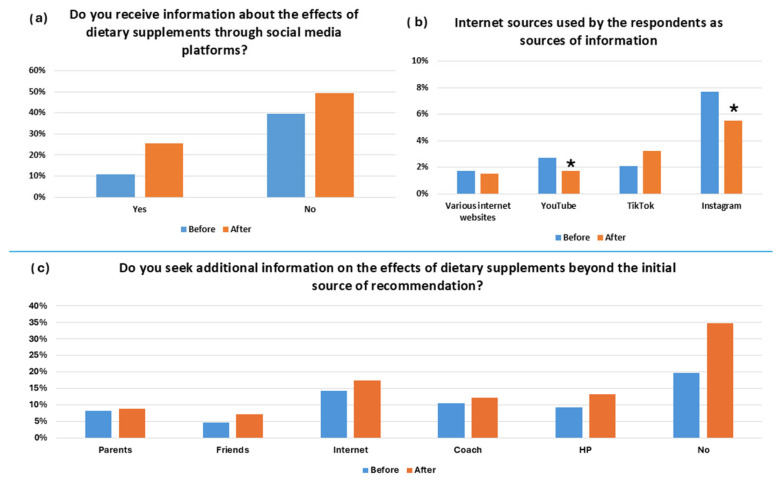
(**a**) Prevalence of social media as a source of information about dietary supplement effects among participants. (**b**) Internet sources used by respondents for information about dietary supplements. (**c**) Proportion of participants who reported seeking further information about dietary supplements beyond their original source of recommendation (HP—healthcare professionals, doctor/pharmacist/nutritionist). Values marked by asterisks (*) indicate statistically significant differences (*p* ˂ 0.01).

The fifth section of the questionnaire addressed the social media influence as a source of information about supplements (Figure 5).

After the intervention, an increase, not statistically significant, was observed in the proportion of respondents who reported receiving information about dietary supplements through social media platforms (~11% before vs. ~26% after).

Instagram was the most widely used platform for supplement information both before (~8%) and after (~6%) the intervention, followed by YouTube (~3% before, ~1.5% after), TikTok (~2% before, ~3% after), and various internet websites (~1.5% before and after). Post-intervention changes were characterized by a decline in YouTube (from ~3% to ~1.5%) and Instagram (from ~8% to ~6%), both of which were statistically significant (*p* < 0.01, YouTube—odds ratio = 0.412; CI = 0.144–1.045, Instagram—odds ratio = 0.400; CI = 0.209–0.730), and a modest increase in TikTok (~2% to ~3%). Usage of various internet websites remained stable (~1.5%).

The proportion of participants seeking additional information did not differ significantly before and after the intervention. Internet was the most commonly cited source (14% to 17%), followed by Coach (10% to 12%), HP (9% to 13%), Parents (8% to 9%), and Friends (4.5% to 7%).

**Figure 6:**
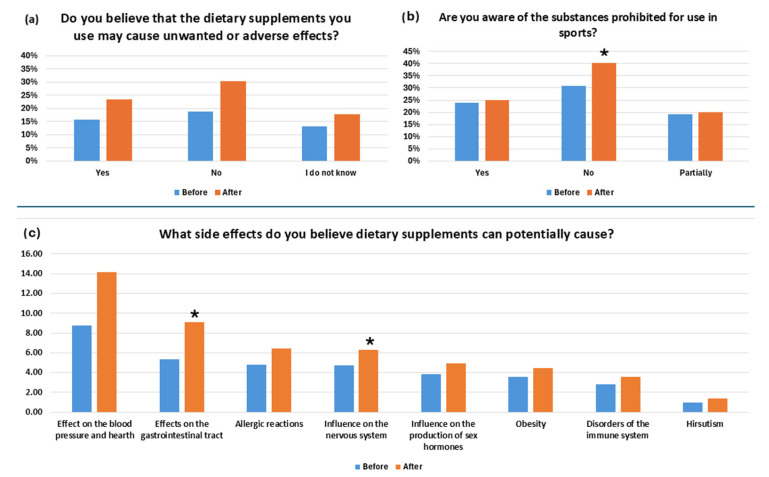
(**a**) Participant perceptions of the potential for unwanted or adverse effects from the dietary supplements they use. (**b**) Participant awareness of substances banned in sports, as related to anti-doping regulations and prohibited substance lists. (**c**) Perceived potential side effects of dietary supplements as reported by participants. Values marked by asterisks (*) indicate statistically significant differences (*p* ˂ 0.05).

The potential adverse effects of supplements and banned substance awareness was surveyed in the sixth section of questions (Figure 6).

Regarding the perceived potential for adverse effects of dietary supplements, increases were observed across all response categories after the intervention. None of the changes detected did not reach statistical significance, suggesting that the intervention did not substantially shift participants’ overall attitudes regarding the potential harms of the supplements they use.

With respect to the awareness of substances prohibited in sports, “Yes” responses remained stable (~23% before vs. ~24% after) and “Partially” was also unchanged (~19% both before and after). However, the proportion of participants reporting no awareness of prohibited substances increased from ~30% to ~39%, a change that was statistically significant (*p* < 0.05, odds ratio = 0.774; CI = 0.580–1.030).

Among perceived potential side effects, effects on blood pressure and the heart were the most commonly reported both before (8.5%) and after (14%) the intervention. Statistically significant increases were observed for effects on the gastrointestinal tract (5.5% to 9%, *p* < 0.05, odds ratio = 0.182; CI = 0.020–0.833) and the nervous system (4.5% to 6%, *p* < 0.05, odds ratio = 0.333; CI = 0.078–1.100). Other reported side effects increased modestly but not significantly: allergic reactions (4.5% to 6%), sex hormone production (4% to 5%), obesity (3.5% to 4.5%), immune system disorders (3% to 3.5%), and hirsutism (1% to 1.5%).

**Figure 7:**
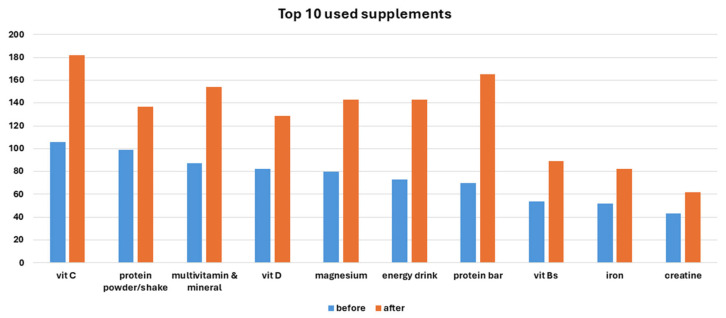
Top 10 dietary supplements most commonly used by participants based on the questionnaire answers.

A graphical representation of the ten most commonly used supplements among the surveyed population is presented in Figure 7. In the survey, a multiple-choice question with multiple permitted responses was employed, offering 25+ supplement options for selection. The prevalence of use was calculated based on the proportion of participants selecting each supplement.

Notably, the same eight supplements remained the most frequently used both before and after the educational intervention. However, a certain increase in the reported use of energy drinks was observed following the intervention. One potential explanation for this rise is the timing of the post-intervention survey, which coincided with final exams and entrance examinations for high schools and universities.

### 3.2. Social Media Intervention

**Figure 8:**
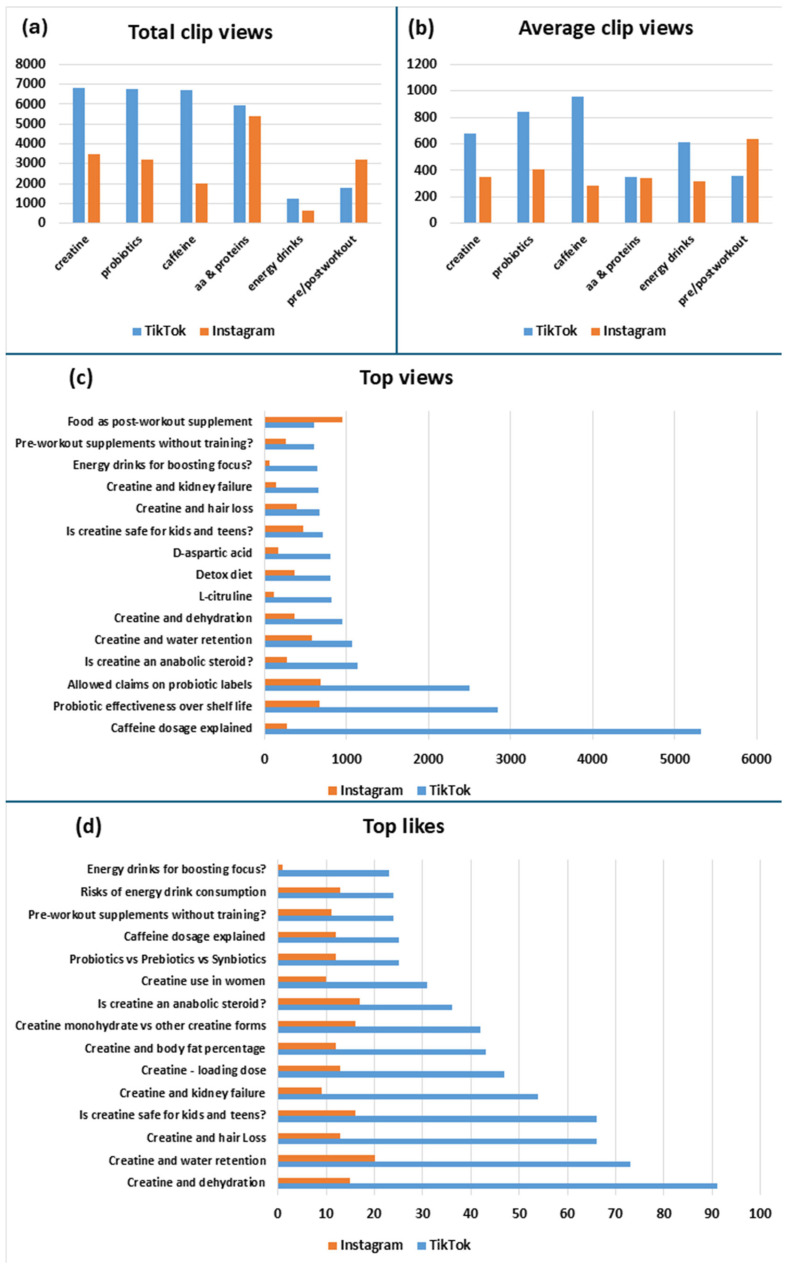
(**a**) Total clip views grouped by categories of dietary supplements. (**b**) Average clip views grouped by categories of dietary supplements. (**c**) Top views for individual videos. (**d**) Top likes for individual videos.

In order to gain insight into the influence of our social media intervention, we have analyzed clip “views” and “likes”, and the results are shown in Figure 8.

In terms of video analytics, TikTok emerged as the leading platform, both in total number of video views (29,178 TikTok vs. 17,935 Instagram) and in average views per video. Among the supplement-related topics, creatine, probiotics, and caffeine were the top-performing keywords, ranking highest in both total and average view counts. The dominance of TikTok in terms of video reach confirms its relevance and effectiveness as a dissemination channel for health-related educational content among adolescents. The high viewership of videos related to creatine, probiotics, and especially caffeine aligns with current trends in supplement interest within this age group.

The video addressing caffeine dosage received the highest number of views overall. However, it did not receive the highest number of likes. The discrepancy between views and likes—particularly in the case of the caffeine dosage video—could be attributed to several factors. One possible explanation is the influence of non-content-related variables, such as the speaker’s perceived “*likeability*” or presentation style. Another plausible reason may be the adolescents’ reluctance to “like” videos on topics perceived as sensitive or potentially controversial, such as caffeine use, due to concerns over leaving a digital trace of their interest.

## 4. Discussion

Educational interventions have been shown to significantly improve knowledge and perceptions regarding dietary supplements among adolescents and young adults. A lecture-based intervention led to statistically significant improvements in students’ understanding of supplement safety and risks [25]. Furthermore, a review of interventional studies reported consistent positive effects on knowledge and attitudes related to supplement use [26]. Although certain educational materials and interventions have been implemented, their availability, quality, and effectiveness remain inconsistent, and their overall impact on knowledge and especially behavior is still limited [14,15,16,26]. In our, study we analyzed the effects of short-term educational intervention, consisting of 56 videos on TikTok and Instagram, thematically divided into six groups (as shown in Figure 8a,b).

In our study, 65% of respondents identified themselves as physically active; however, only about 20% reported engaging in physical activity on a daily basis. These data are consistent with findings from the literature [27,28]. This means that only one in five adolescents in Serbia meet the World Health Organization (WHO) recommendation of 60 min of moderate-to-vigorous physical activity per day [29,30].

The most common form of physical activity was the gym, followed by participation in team sports, primarily football and volleyball, along with basketball. These findings partially align with the results of study conducted in Slovenia, where the majority of adolescents were engaged in strength and power sports—reported by as many as 89% of male and 48% of female participants. Similarly, football and basketball were identified as the most popular team sports in that study [30].

### 4.1. Dietary Supplement Use in Serbian Adolescents

Findings from our study show that the majority of adolescents initiate dietary supplement use independently, without prior consultation with professionals or adults, which is consistent with the patterns observed in a similar study conducted in Slovenia. In the Slovenian study, between 26% and 49% of adolescents reported starting supplement use on their own, depending on gender and level of physical activity [30].

Surveys in Serbia confirm that many athletes and recreational athletes start using dietary supplements without consulting healthcare professionals. A study among students in the city of Niš (Serbia) reported that over half of the participants were inadequately informed on dietary supplement use, citing media and peers as their primary sources of information [12]. Similarly, for the majority of students in Belgrade, most users acquired information about supplements through informal channels (media, friends), rather than through professional advice [13]. In one cross-national study involving 348 young athletes from Serbia, Germany, Japan and Croatia competing in 18 sports at the international level, coaches were identified as the primary source of information on supplementation, while consultations with healthcare professionals were rare. Alarmingly, less than 40% of participants had accurate knowledge about the proper and intended use of protein, creatine, amino acids, beta-alanine and glutamine, although their understanding of vitamins and minerals, sports drinks and caffeine was higher [14].

The results of our study identified the ten most commonly used dietary supplements among adolescents in Serbia, with a clear predominance of vitamins and minerals. This finding aligns with global trends observed in similar populations, suggesting a consistent pattern in supplement use during adolescence. Vitamins such as vitamin C, vitamin D, vitamin Bs and multivitamin complexes, along with minerals like magnesium and iron, were among the most frequently reported in our sample. When compared to previous research, our findings show notable similarities. Studies conducted in the United States have also highlighted vitamins and minerals as the primary supplements used by adolescents [31,32]. From a safety perspective, the majority of the supplements among the top 10 in Figure 7 do not raise much concern (multivitamins, vitamin C, D and B, protein powder and bars, and magnesium), since they are generally considered safe for use, even in minors. Of note, however, are high-ranking energy drinks (ED). While not traditional dietary supplements, energy drinks are often grouped into the broader category of ergogenic or performance-enhancing products and are widely marketed to adolescents. In this high-ranking perspective, our findings warrant certain considerations. First of all, the study population consisted of teens 14–18-year-old, out of which the majority (92.6%) were minors (14–17 y) (Figure 1). Secondly, more than half of the respondents (64.1%) practice sport regularly, and an additional 13.9% practice it occasionally (Figure 3). Taken together, these data may suggest the inclination of minor athletes to use energy drinks. Nevertheless, further research is needed for a definitive conclusion.

### 4.2. Social Media Intervention Impact

Based on the comparison of the questionnaire answers pre- and post- intervention (Figure 4, Figure 5 and Figure 6), it is evident that our social media intervention had modest impact. Although designed as a pilot study, with limited duration and overall reach, several points are important to note. After the intervention, a decrease in the use of Internet forums (Figure 4c) and YouTube and Instagram (Figure 5b), as a source of information for supplement use, was detected. Furthermore, there was an increase in the notion of a lack of knowledge regarding prohibited substances in sport (Figure 6b). In addition, the percentage of participants who buy supplements in pharmacies rose significantly after the intervention (Figure 4b). Although these findings may not be exclusively attributed to the effect of our social media campaign, they may indicate a certain influence on adolescent behavior. However, based on the number of “views and likes” of the produced short videos (Figure 8), social media educational material may be considered as an effective way to deliver concise but reliable information to adolescents. To obtain stronger impact, the “intervention” should be consistently delivered to the targeted population during a longer time span (>6 months). It is important to emphasize that the overall effect of the various social media interventions aiming to educate on nutrition was positive [33,34,35,36]. Although there are various literature data on the use of social media for nutritional education, the results are difficult to compare due to a lack of uniformity. General rules and guidance, to be defined internationally, are warranted for the future.

Our results also indicate that an important topic for future educational efforts should cover energy drink (ED) consumption in minors (adolescents in particular), given the literature data [37,38,39,40,41,42,43] regarding the alarming effects they produce in children and minors. Although energy drinks are not supplements by definition, they are marketed as ergogenic aids and frequently used to enhance physical performance in physically active adolescents (young athletes).

## 5. Conclusions

Our study provided certain insights into the adolescent behavior regarding supplement use and physical activity, which follow general European trends. In addition, it showed that an educational campaign may be conducted using social media platforms (primarily TikTok and Instagram); however, it should last longer than a six-month period, in order to produce a more significant impact. In our view, further educational efforts on social media should be aided by a set of general rules and guidelines that are generated internationally.

## Data Availability

The original contributions presented in this study are included in the article material. Further inquiries can be directed to the corresponding author.

## Abbreviations

The following abbreviations are used in this manuscript:

ADEL	Anti-Doping Education and Learning
CAGR	Compound Annual Growth Rate
COVID-19	Coronavirus Disease 2019
WADA	World Anti-Doping Agency
ED	Energy Drinks
WHO	World Health Organization
